# Fracture of the Superior Maxillary Bone

**Published:** 1869-11

**Authors:** R. H. McKay

**Affiliations:** U. S. A.


Fracture of the Superior Maxillary Bone.—By Dr. R. H. McKay, A. A. S., U. S. A.—Prince Walker, a private of "I" detachment, 38th U. S. Infantry (colored troops), on duty at Fort Craig, New Mexico, was admitted into hospital August 24th, 1869, for injuries received by a hatchet or small "hand ax," in the hands of one of his comrades. He was lying on his bunk at the time—feeling as he said quite unwell. His comrade and he had been quarreling that morning, in the course of which the patient accused the other of stealing. It appears that he brooded over the insult until he arrived at the determination to be revenged in a very summary manner, and to this end rushed into the quarters where the patient was lying, and struck him a powerful blow, evidently aiming for the head, but, probably, by the exertions of the patient to avoid the stroke, missed his mark, and struck him on the face; members of the company then interfered, and prevented a repetition of the blow.

The patient was brought to hospital, his wound washed and examined; the corner of the ax had grazed the frontal bone over the minor angle of the orbit, then descending upon the face quite close to the base of the nose, caused a very considerable flesh wound of the face. The examination was then continued to the inside of the mouth, when the full extent of the injury was more apparent. The bone was observed to be fractured from a point commencing between the central incisors in front, extending thence directly back along the course of the suture, between the maxillary bones, for about one inch in extent, thence curving backwards and outwards, terminated between the last bicuspid and first molar teeth.

This whole piece, including the teeth, was forced down into the mouth, the posterior portion of it a full half inch below its normal position, while the anterior portion was displaced to about half that extent. I experienced considerable difficulty in replacing the fractured portion, but after several unsuccessful efforts, succeeded so perfectly as to justify the belief that it would remain so without artificial means for its support.

The treatment consisted of cold water locally, until inflammatory action had ceased, then simple cerate dressing, observing the usual precautions of keeping the wound thoroughly cleansed. This, with perfect rest of the jaws, except so much as was necessary in taking liquid food, constituted the entire course of treatment. The fracture in the roof of the mouth healed very rapidly, so much so that in a few weeks time only a cicatrix served to mark the line of fracture. The wound upon the face still continues discharging a small amount of pus, with occasional spiculæ of bone, but the patient has so far recovered as to perform duty as cook for the company, and only complains of want of sensation of the fractured part, which is not to be wondered at when we consider the amount of displacement that existed at the time of the injury. I think, too, that the discharge from the face is likely to continue for some little time, as of necessity there must be a very considerable amount of detached pieces of bone yet to come away; yet the progress which the case has made thus far encourages the belief that perfect recovery, with perfect adaptability and use of the injured parts will speedily ensue.

